# Spatial Succession Underlies Microbial Contribution to Food Digestion in the Gut of an Algivorous Sea Urchin

**DOI:** 10.1128/spectrum.00514-23

**Published:** 2023-04-25

**Authors:** Matan Masasa, Ariel Kushmaro, Dzung Nguyen, Helena Chernova, Nadav Shashar, Lior Guttman

**Affiliations:** a Marine Biology and Biotechnology Program, Department of Life Sciences, Ben-Gurion University of the Negev, Eilat, Israel; b Israel Oceanographic and Limnological Research, The National Center for Mariculture, Eilat, Israel; c Avram and Stella Goldstein-Goren Department of Biotechnology Engineering, Ben-Gurion University of the Negev, Beer-Sheva, Israel; d The Ilse Katz Center for Nanoscale Science and Technology, Ben-Gurion University of the Negev, Beer-Sheva, Israel; e School of Sustainability and Climate Change, Ben-Gurion University of the Negev, Beer-Sheva, Israel; f Ben-Gurion University of the Negev, Department of Life Sciences, Beer-Sheva, Israel; Lerner Research Institute

**Keywords:** gut microbiome, host-bacterium associations, microbial ecology, niche specification, algal diet, metabolism, metagenomics, sea urchins

## Abstract

Dietary influence on the microbiome in algivorous sea urchins such as Tripneustes gratilla elatensis suggests a bacterial contribution to the digestion of fiber-rich seaweed. An ecological insight into the spatial arrangement in the gut bacterial community will improve our knowledge of host-microbe relations concerning the involved taxa, their metabolic repertoire, and the niches of activity. Toward this goal, we investigated the bacterial communities in the esophagus, stomach, and intestine of *Ulva*-fed sea urchins through 16S rRNA amplicon sequencing, followed by the prediction of their functional genes. We revealed communities with distinct features, especially those in the esophagus and intestine. The esophageal community was less diverse and was poor in food digestive or fermentation genes. In contrast, bacteria that can contribute to the digestion of the dietary *Ulva* were common in the stomach and intestine and consisted of genes for carbohydrate decomposition, fermentation, synthesis of short-chain fatty acids, and various ways of N and S metabolism. *Bacteroidetes* and *Firmicutes* were found as the main phyla in the gut and are presumably also necessary in food digestion. The abundant sulfate-reducing bacteria in the stomach and intestine from the genera *Desulfotalea*, *Desulfitispora*, and *Defluviitalea* may aid in removing the excess sulfate from the decomposition of the algal polysaccharides. Although these sea urchins were fed with *Ulva*, genes for the degradation of polysaccharides of other algae and plants were present in this sea urchin gut microbiome. We conclude that the succession of microbial communities along the gut obtained supports the hypothesis on bacterial contribution to food digestion.

**IMPORTANCE** Alga grazing by the sea urchin *Tripneustes gratilla elatensis* is vital for nutrient recycling and constructing new reefs. This research was driven by the need to expand the knowledge of bacteria that may aid this host in alga digestion and their phylogeny, roles, and activity niches. We hypothesized alterations in the bacterial compositional structure along the gut and their association with the potential contribution to food digestion. The current spatial insight into the sea urchin’s gut microbiome ecology is novel and reveals how distinct bacterial communities are when distant from each other in this organ. It points to keynote bacteria with genes that may aid the host in the digestion of the complex sulfated polysaccharides in dietary *Ulva* by removing the released sulfates and fermentation to provide energy. The gut bacteria’s genomic arsenal may also help to gain energy from diets of other algae and plants.

## INTRODUCTION

The gut microbial community of many animals contributes various services for the host in food digestion, health maintenance, and immune response regulation (1). Algivorous sea urchins like Tripneustes gratilla elatensis are essential for nutrient recycling along the food web in their habitats (2, 3). Moreover, this species’ algal grazing from the rocky substrate is vital for coral larvae to settle as pioneers in newly established reefs (4). Unlike other echinoderms (*Asteroidea*, *Crinoidea*, *Ophiuroidea*, or *Holothuroidea*), this sea urchin is strictly algivorous (5). This opens the window for studies on the associations between such a diet and the gut microbiome. The tubular alimentary canal is its largest and longest organ, wrapped in a complex multiloop structure between the mouth and anus at the top and bottom sides of the body, respectively. It consists of three major parts: the esophagus (closest to the mouth), stomach (median), and intestine (closest to the anus). Various characteristics differ between these three regions that make them easily identifiable. The esophagus is short, thin, and brownish; the stomach is long, thick, and bright, and has a fleshy texture; and the intestine is dark and short (6).

Food transport downstream in the gut is generated by upstream pressure from newly ingested food and requires defecation rapidly (6, 7). As the gut does not consist of anatomical barriers between the different regions (6), older and newer food is stirred together when new food enters the gut and digesta is excreted. The digestive system can function as a continuous-flow, stirred-tank reactor (8). The entire transit of food (from the mouth to the anus) is aided by peristaltic contractions of muscles in the gut wall and the beating of luminal cilia (6, 7) and may take hours or days, with the shortest retention time in the esophagus (between 1 and 2 h) and the longest in the intestine (between 12 and 48 h). Thus, much food digestion occurs in the downstream regions (8). Functionality-wise, cells in the esophagus are in charge of mucous secretion for wrapping the ingested food into pellets which then transit along the digestive tract (9). The stomach’s primary function is in the secretion of digestive enzymes, while nutrient absorption occurs primarily in the intestine (10). Compared to other regions, the stomach reveals relatively extreme conditions with high acidity (pH ~4.5, compared to pH ~7.2 in the other parts), oxygen depletion (between 0 and 40 μM at a distance of 50 μm from the gut wall), and CO_2_ richness (two-times-faster production than that in the intestine) (8, 11).

From a microbial ecology point of view, the relatively extreme conditions in the stomach may produce a robust deterministic force on the resident bacterial community that will influence its composition and perhaps also its functions. As the longest region in the gut, it can also serve as an ecological barrier between the upstream esophagus and downstream intestine which may result in different bacterial communities in these distal ends of the gut (11). Functionality-wise, spatial alterations in the composition and functions of the gut bacteria may point to a succession process where communities further down depend on the initial degradation steps performed by the communities further up. Compared to deposit-fed sea urchins, the algal diet of *T. gratilla elatensis* is more complex as it requires digestion of the cell wall polysaccharides (12, 13). The low capacity of endogenic digestive enzymes (14) and the high dietary fiber content in the algal diet seem challenging for energy gain (15). Previous studies proposed bacterial contribution in food digestion in various sea urchin species (16–19). A recent study revealed that different algal diets induced alteration in the composition of the gut microbial community of *T. gratilla elatensis* (18). It was shown that dietary Ulva fasciata improved the growth of *T. gratilla elatensis* while developing a unique microbial community with unique associations between networking microbes (18). Still, more knowledge is required regarding the potential metabolisms by which gut bacteria contribute to *Ulva* digestion and the regions where such metabolisms occur. The ability of *T. gratilla elatensis* to feed solely on *Ulva* is a significant advantage in examining the potential contribution of the gut bacteria in feed digestion and the sites where different metabolisms occur. Toward this goal, we used high-throughput metagenomics analyses to examine the composition and potential functions of the bacteria along the gut of this sea urchin when fed on *Ulva fasciata.*

## RESULTS

### Spatial differentiation and niche-specific microbes in the gut bacterial community.

**FIG 1:**
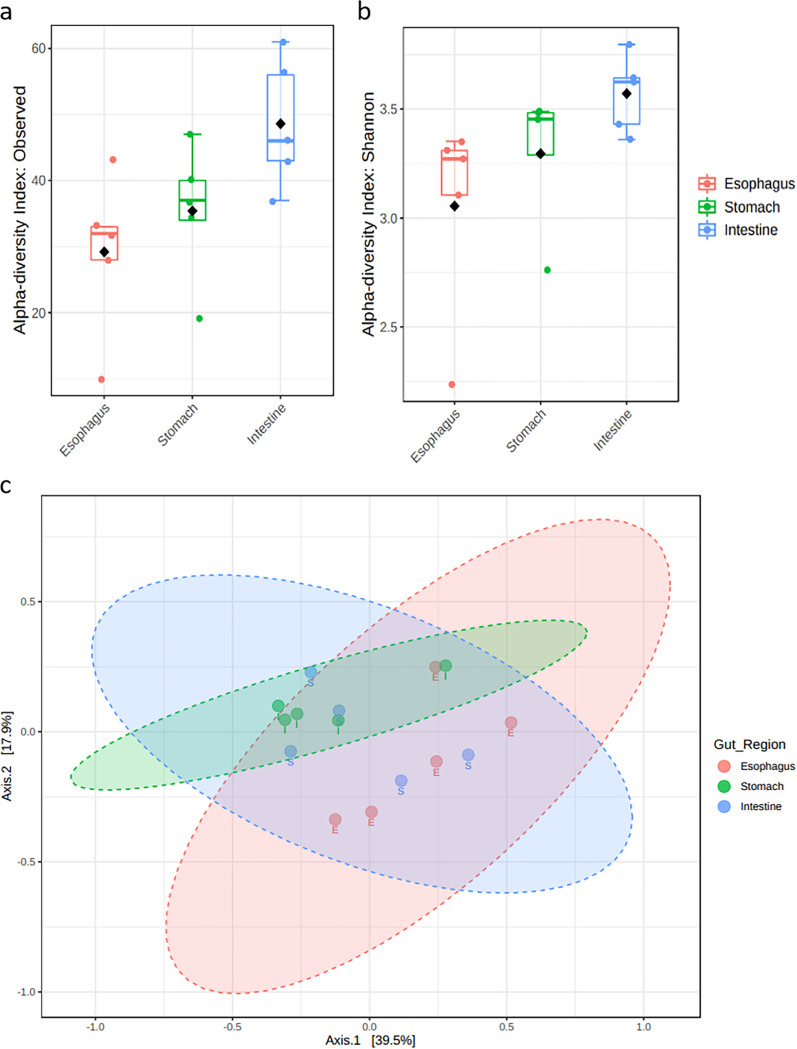
Ecological indices of richness and diversity of the microbial assemblies in the gut of *T. gratilla elatensis*. (a and b) Richness (a) and Shannon diversity (b) indices are presented for the different gut regions of esophagus, stomach, and intestine. Box plots represent the variation between the five biological replicates within each examined gut region. Black diamonds mark mean values; medians and standard deviations (SD) are shown within and outside the box plots, respectively. (c) The dissimilarity between the microbial assemblies in the different examined gut regions is demonstrated by a principal-coordinate analysis (PCA) generated based on Bray-Curtis dissimilarity (*n* = 15).

A total of 40,969 high-quality reads were extracted from the data across 15 samples. The library size ranged between 1,167 and 4,223 reads per sample and clustered into 329 unique amplicon sequence variants (ASVs) (see Fig. S1 in the supplemental material). Rarefaction curves based on alpha diversity revealed a minimum library size of 1,167 ASVs as the threshold for a regional effect on community richness (*H*_14,3_ = 6.4179; *P *< 0.05; Fig. S2). A spatial differentiation was significant only between the intestinal and esophageal microbial communities. Richness was highest in the intestine and lowest in the esophagus with 48.6 ± 9.76 and 29.2 ± 12.07 ASVs, respectively (Fig. 1a). The intestinal microbial community was also more diverse than that in the esophagus (*H*_14,3_ = 7.22; *P* < 0.05; Fig. 1b). However, these spatial differentiations were not significant when comparing the microbial community in each of these end regions of the gut with that in the intermediate stomach region (*F*_14,3_ = 1.3514, *P* < 0.21, *R*^2^ = 0.18, stress = 0.094; Fig. 1c).

**FIG 2:**
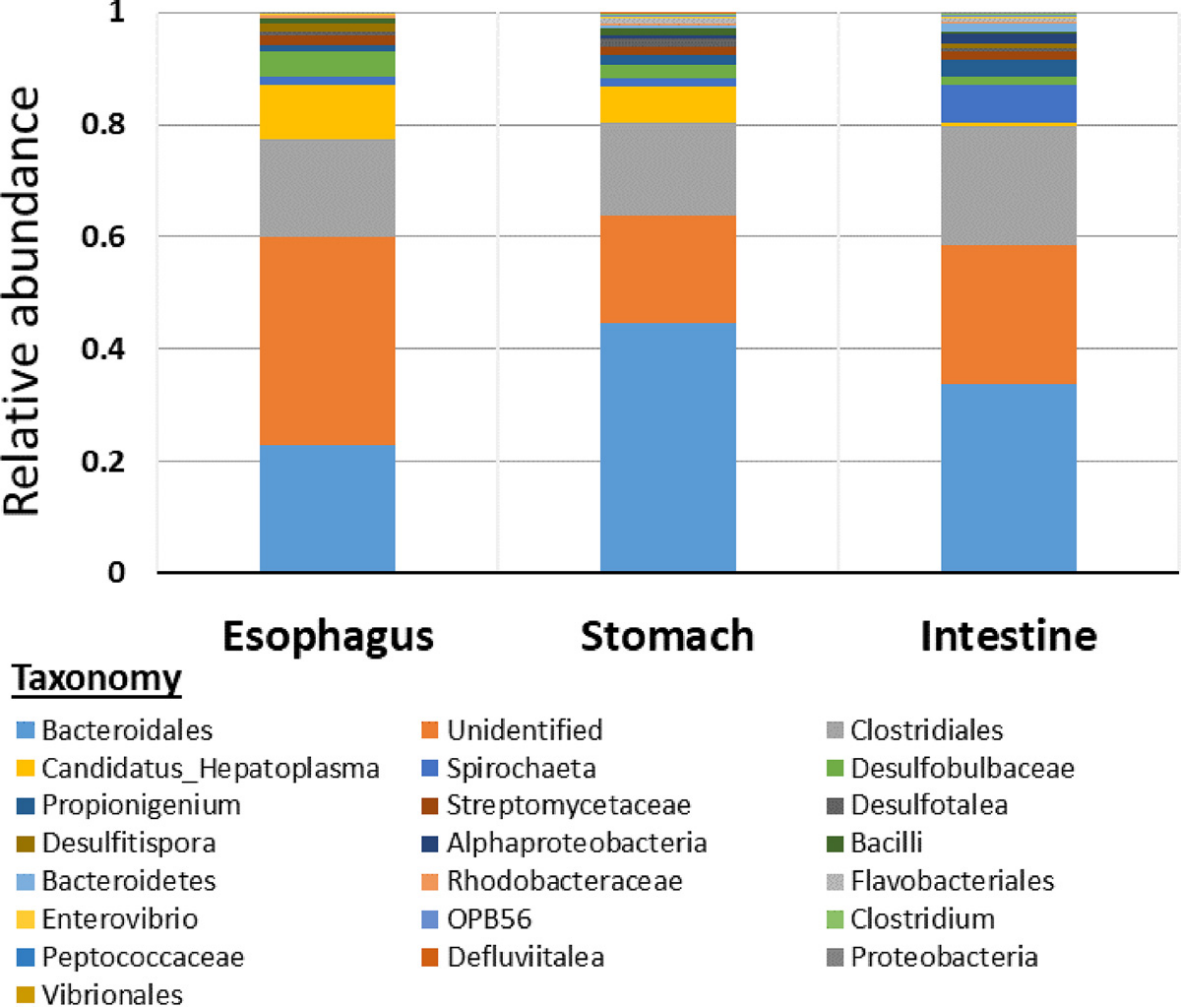
Relative abundances of the various bacterial taxa in the gut assemblies of *T. gratilla elatensis*. For each taxon, mean relative abundance (in percent) was calculated for each gut region based on the five examined biological replicates (out of the total of 15 samples). The taxonomic classification of ASVs was set (at the highest taxonomic level).

**FIG 3:**
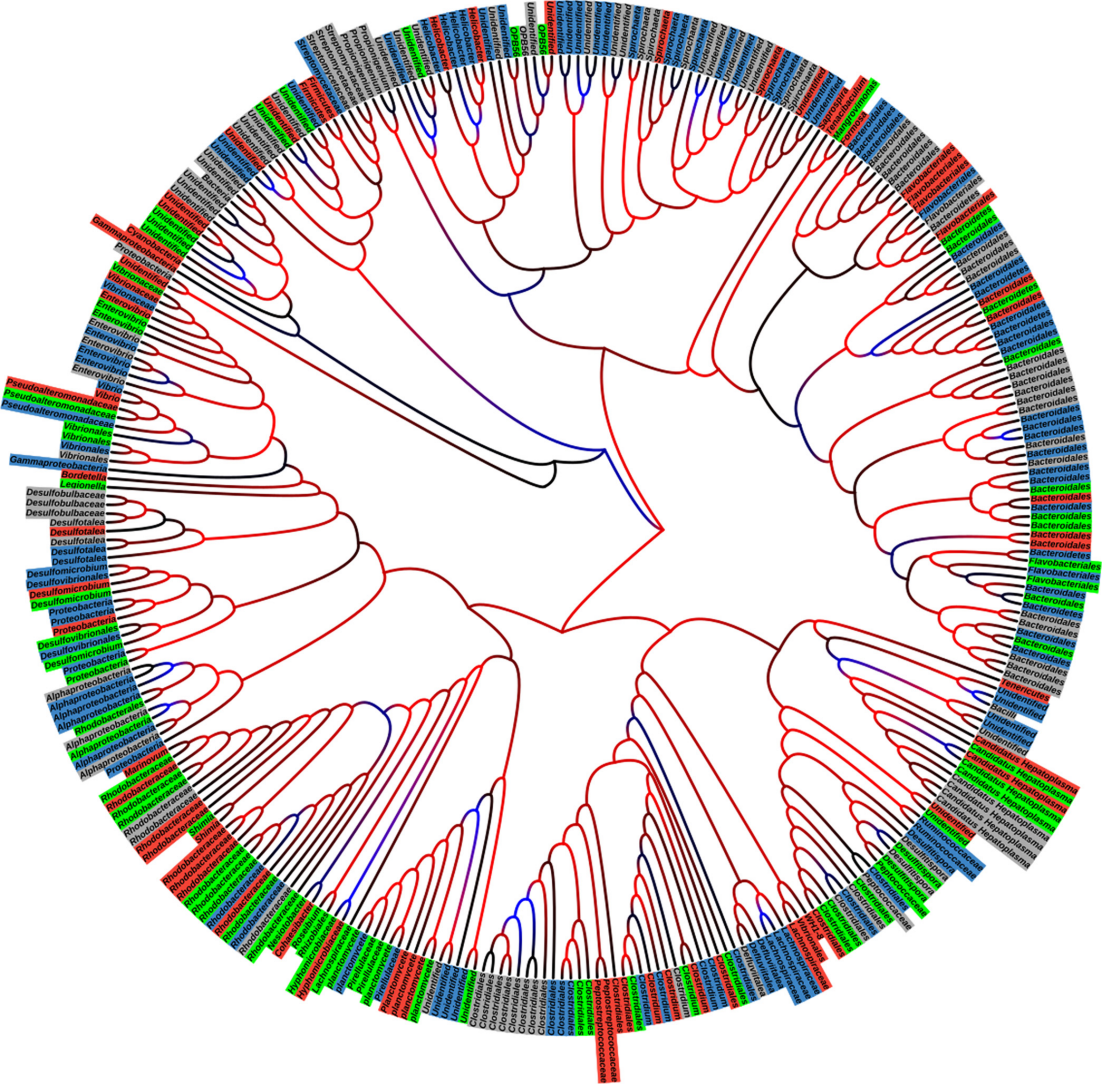
Phylogenetic tree of *T. gratilla elatensis* gut microbial assembly. ASVs were colored based on their spatial niche specification in the gut as follows: the red ASVs were exclusive to the esophagus, the green ASVs were exclusive to the stomach, and the blue ASVs were exclusive to the intestine. Gray ASVs occurred in at least two different gut regions. The phylogenic tree of closeness between the various ASVs was constructed using the Interactive Tree of Life (iTOL) tool based on the weighted UniFrac indices. The branch was colored based on their bootstraps (1,000); the minimum was represented in blue, the midpoint in black, and the maximum in red.

The gut microbes were clustered into 22 taxa (Fig. 2). The phylum *Bacteroidetes* dominated the microbial community in the entire gut and each region. The prevalence of this phylum was highest in the stomach (44.5%) and lower in the intestine (35.2%) and esophagus (22.7%). The genus “*Candidatus* Hepatoplasma” showed a decreasing prevalence trend from the esophagus to the intestine, with a relative abundance of 9.6% in the esophagus that decreased along the posterior regions to 6.3% in the stomach (not significantly different from the other regions) and 0.4% in the intestine (significantly different from the esophagus; *H*_9,2_ = 7.71, *P* < 0.05). Compared to other gut regions, *Spirochaeta* was the fourth most abundant taxon in the intestine (6.8%), while its prevalence in anterior regions was significantly lower, below 1.66% (*H*_14,3_ = 8.15, *P* < 0.05). Family *Clostridiales* revealed a relatively similar distribution along the gut with relative abundance between 17 and 21%. The taxa *Proteobacteria*, *Vibrionales*, and *Desulfitispora* were absent from the stomach, while *Alphaproteobacteria*, *Flavobacteriales*, OPB56, *Peptococcaceae*, and *Defluviitalea* were absent from the esophagus. We revealed unique taxonomic groups and niche-specific ASVs that occurred in only one gut region (Fig. 3). From a regional perspective, the esophagus consisted of 68 niche-specific ASVs that were not obtained in the other examined regions, while the number of niche-specific ASVs in the stomach and intestine was 64 and 96, respectively. Out of the niche-specific ASVs in the esophagus, 9 ASVs were from unique taxonomic groups (5 genera, 3 families, and 1 order) that were absent in the other two regions. Unique taxonomic groups in the stomach and intestine consisted of 7 (4 genera, 1 family, 1 order, and 1 phylum) and 1 (family), respectively (Fig. 3). Many of the niche-specific ASVs in the gut were from *Bacteroidales* (32 ASVs), *Clostridiales* (17 ASVs), *Rhodobacteraceae* (16 ASVs), *Spirochaeta* (9 ASVs), and “*Candidatus* Hepatoplasma” (5 ASVs). Despite such unique taxonomic groups and niche-specific ASVs in each of the examined regions, the structure of the bacterial communities in the different regions (i.e., richness, diversity, and composition) was significantly different only between the esophagus and intestine.

### Potential functions and metabolism of the gut bacteria.

**FIG 4:**
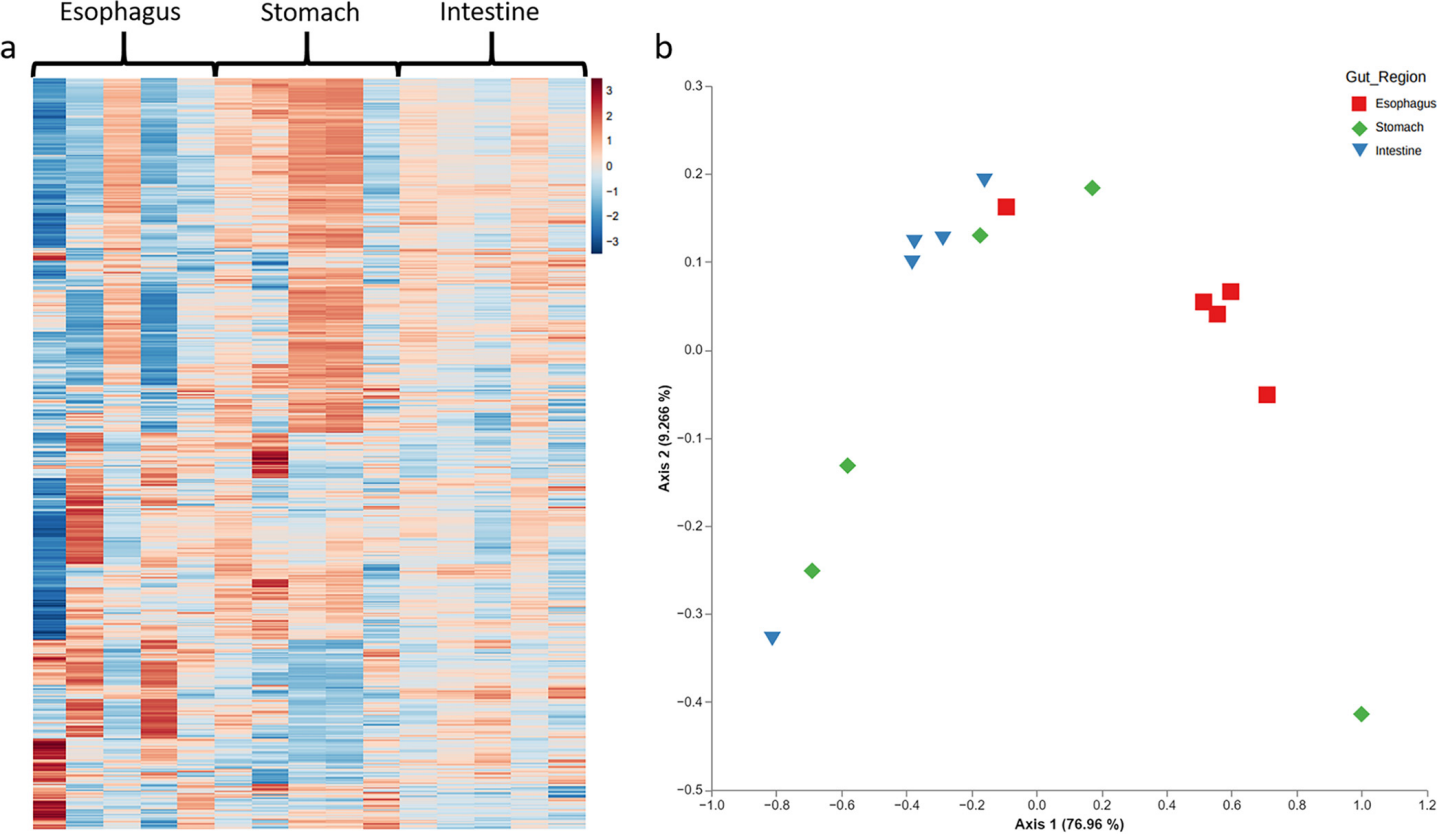
A comparative analysis of the KOs in the gut microbial assemblies of *T. gratilla elatensis*. (a) A heatmap diagram of the KEGG orthologous genes in the three main gut regions of the esophagus, stomach, and intestine. Each gene orthologue is colored according to its number of KOs in each of the five biological replicates (for each gut region); color varies from dark blue (0) to dark red (20,098). (b) A principal-coordinate analysis demonstrates the dissimilarity between the microbial assemblies in the different gut regions regarding their potential functions based on the measured, weighted UniFrac indices (*n* = 15).

The functional analysis identified 30,888,907 reads that may be annotated to known functional genes (an average of 2,059,260 per sample). Reads clustered in 9,027 predicted KEGG Orthologues (KOs), presenting spatial differences in occurrence corresponding to the number of copies (Fig. 4a). Alpha Shannon diversity of the bacterial KOs was relatively similar between the different gut regions (*H*_14,3_ = 3.78, *P* = 0.15, Fig. S3). However, a significant dissimilarity was measured between the different gut regions regarding their content of functional genes (*T *= 3.37, *P* < 0.05, Fig. 4b). The esophagus and the intestine differed significantly (*F*_9,2_ = 11.01, *P* < 0.05), but neither differed significantly from the gene assembly of the stomach community in closest proximity to it (*P* > 0.05).

**FIG 5:**
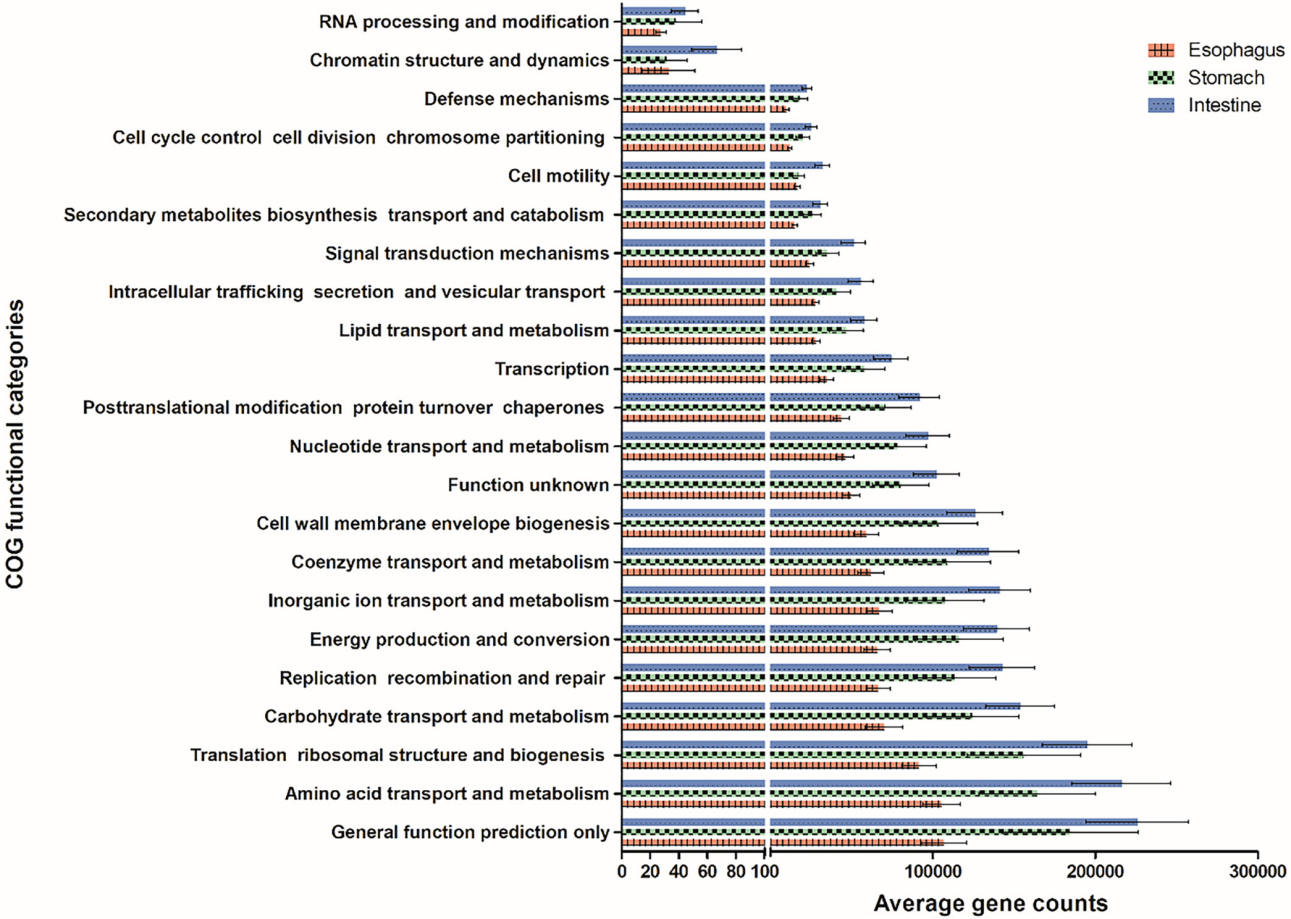
Microbial functional gene distribution in the different gut regions of *T. gratilla elatensis*. The rows represent the number of gene copies (log transformed) in each functional category (annotated by COG) in the esophagus (red), stomach (green), and intestine (blue). Values are means ± SDs (*n* = 15).

A total number of 4,786,473 potential functional genes were sorted into 22 categories (Fig. 5). The most abundant categories were the general functions of amino acid transport and metabolism (485,517), translation, ribosomal structure, and biogenesis (442,412), and carbohydrate transport and metabolism (348,322). The intestine and esophagus differed significantly in their COG (Clusters of Orthologous Groups) content (Table S1), with the intestine leading in the number of genes associated with general functions (*P* < 0.001), amino acid transport and metabolism (*P* < 0.001), inorganic ion transport and metabolism (*P* < 0.001), translation, ribosomal structure, and biogenesis (*P* < 0.01), carbohydrate transport and metabolism (*P* < 0.05), replication, recombination, and repair (*P* < 0.05), energy production and conversion (*P* < 0.05), and coenzyme transport and metabolism (*P* < 0.05). The stomach was also more abundant than the esophagus in genes of the general function category (*P* < 0.05). Still, no significant difference was measured between the stomach and the intestine in any category (*P* > 0.05). Genes of the category RNA processing and modification and the category chromatin structure and dynamics were found in the microbial community in the different gut regions but at a relatively low content of between 1 and 100 copies.

**FIG 6:**
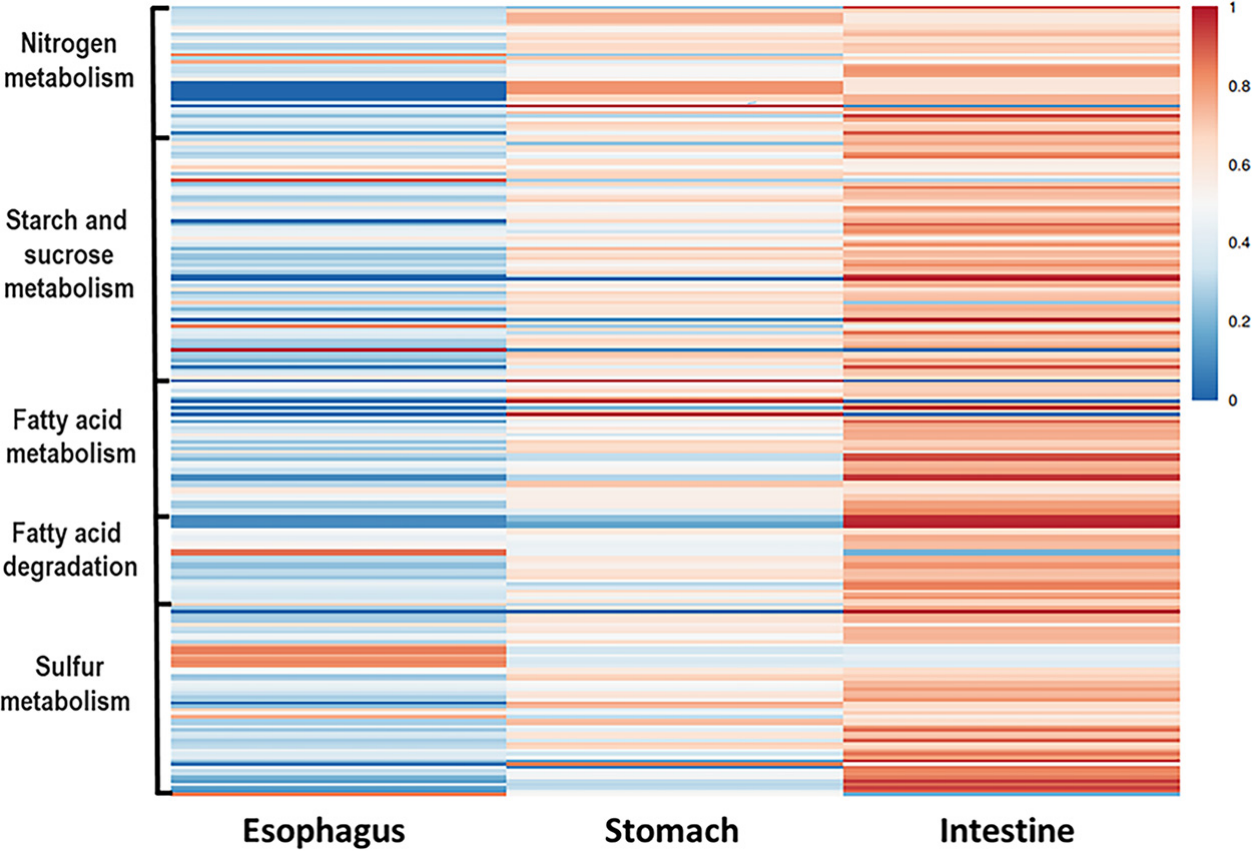
A heatmap diagram of selected KEGG orthologous genes of the microbial assemblies in the different gut regions of *T. gratilla elatensis*. The map highlights the occurrence and the cumulative number of copies after vector scaling in the microbial assembly of the esophagus, stomach, and intestine gut regions.

**FIG 7:**
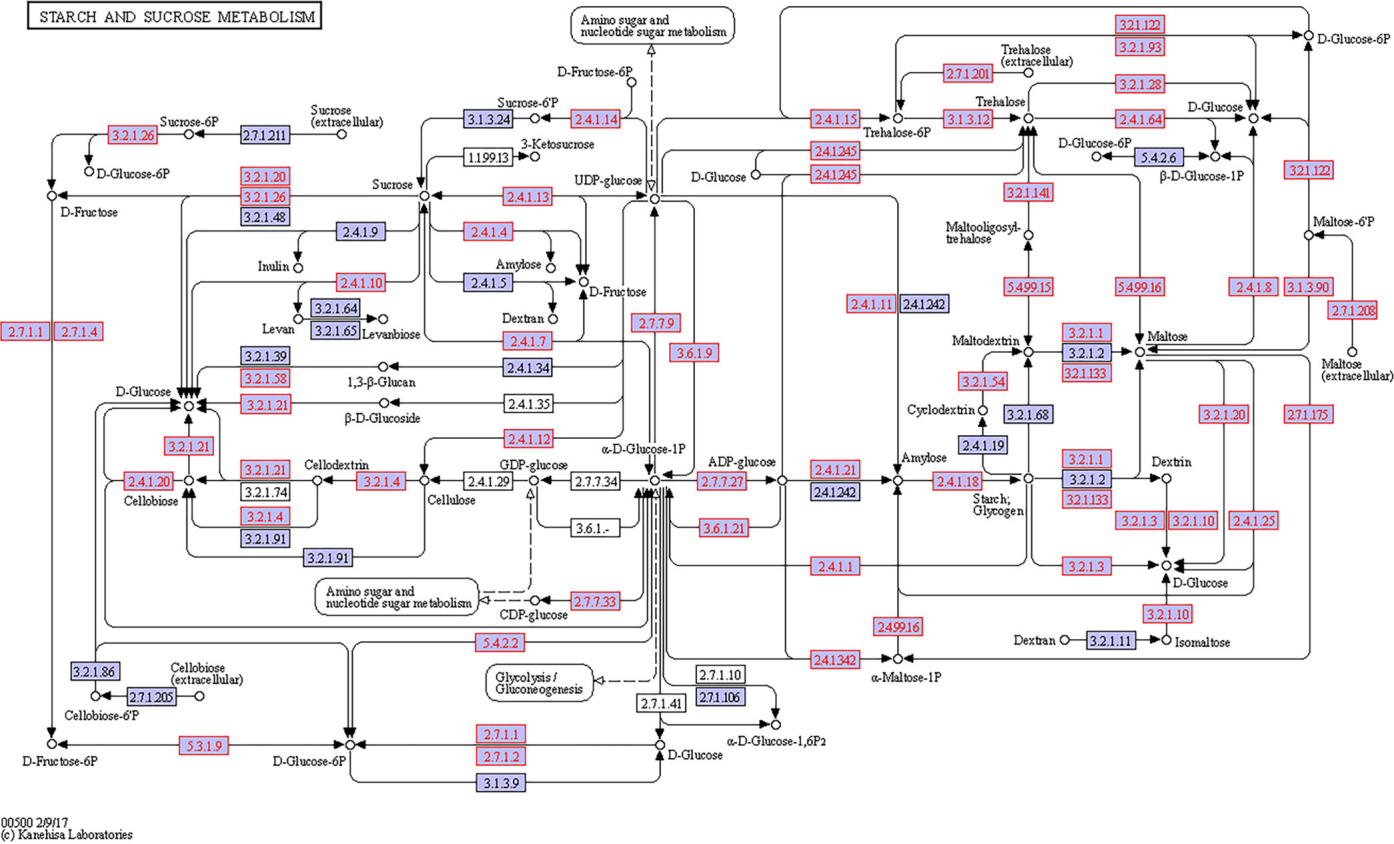
Hypothetical pathway for the starch and sucrose metabolism of the gut microbial assembly of *T. gratilla elatensis*. The metabolic mapping was performed according to KEGG. Purple boxes with red font represent the genes that have been identified, while purple boxes with black font represent other known genes in the hypothetical pathway of each compound (marked by circles). Arrows present the direction of the metabolic function.

**FIG 8:**
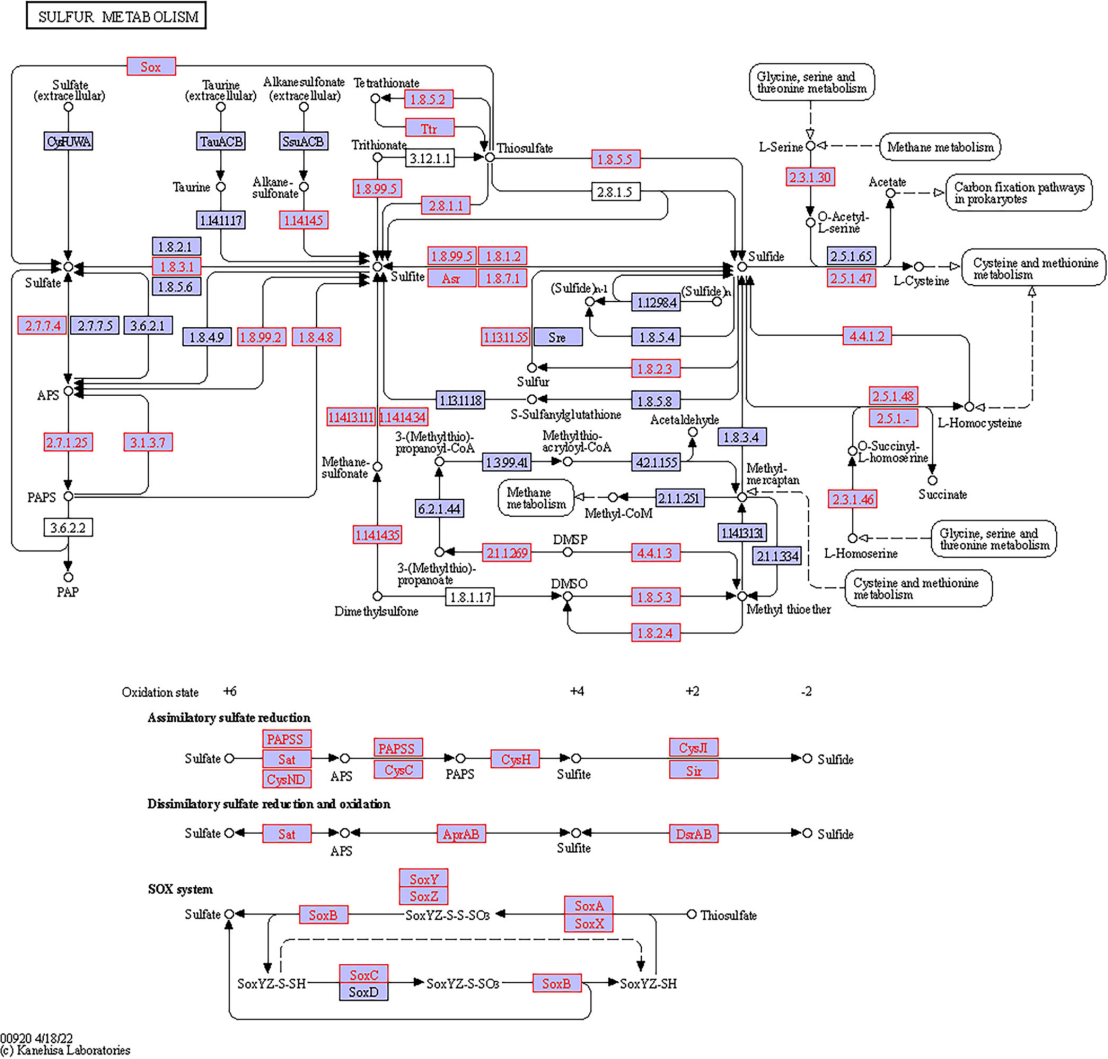
Hypothetical pathway for the sulfur metabolism of the gut microbial assembly of *T. gratilla elatensis.* The metabolic mapping was performed according to KEGG. Purple boxes with red font represent the genes that have been identified, while purple boxes with black font represent other known genes in the hypothetical pathway of each compound (marked by circles). Arrows present the direction of the metabolic function. Abbreviations: APS, adenosine 5′-phosphosulfate; PAP, phosphoadenosine phosphate; PAPS, 3′-phosphoadenosine 5′-phosphosulfate; CoA, coenzyme A; CoM, coenzyme M; DMSO, dimethyl sulfoxide; DMSP, S-dimethylsulfonium propionic acid.

**FIG 9:**
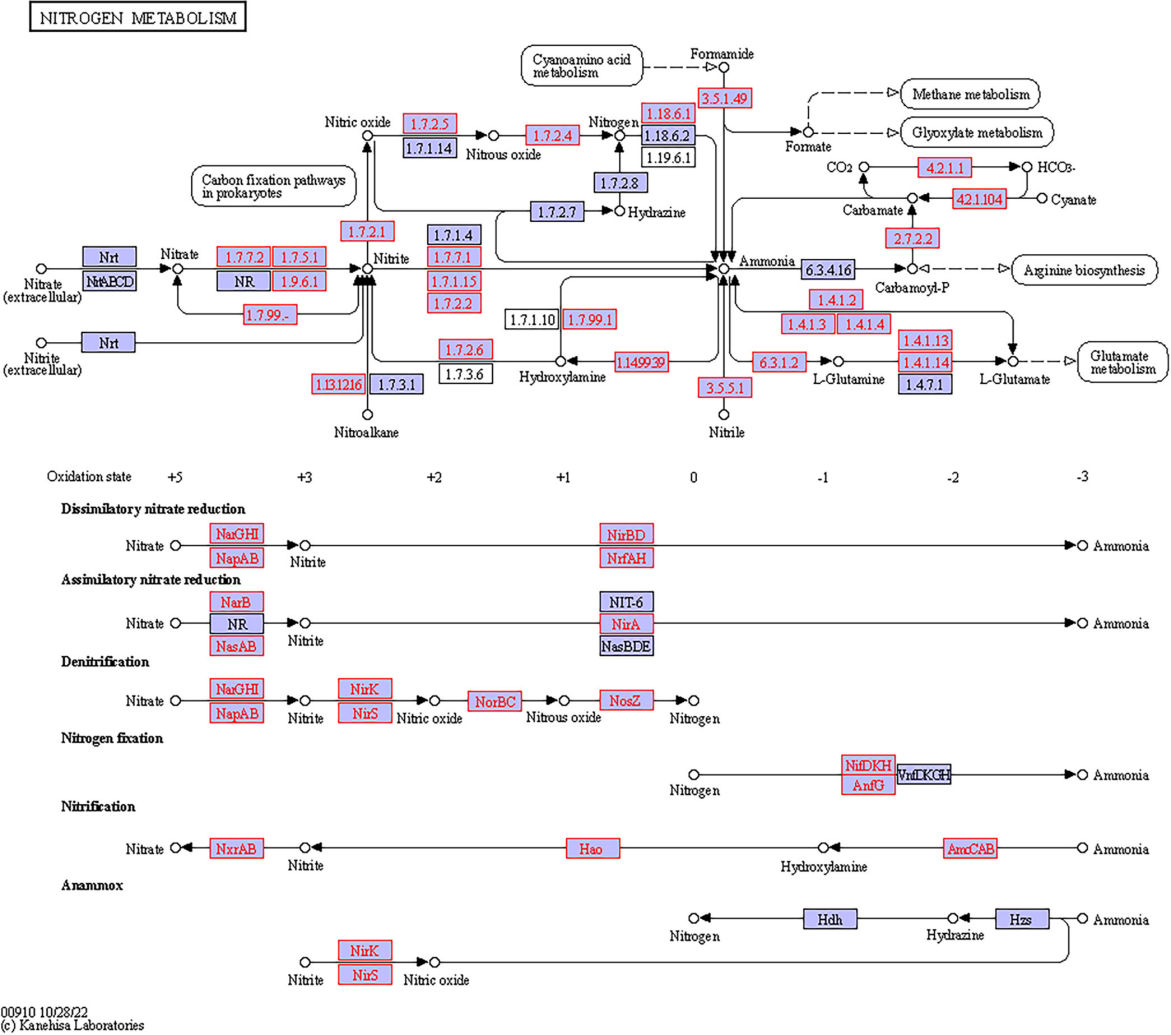
Hypothetical pathway for the nitrogen metabolism of the gut microbial assembly of *T. gratilla elatensis*. The metabolic mapping was performed according to KEGG. Purple boxes with red font represent the genes that have been identified, while purple boxes with black font represent other known genes in the hypothetical pathway of each compound (marked by circles). Arrows present the direction of the metabolic function.

**FIG 10:**
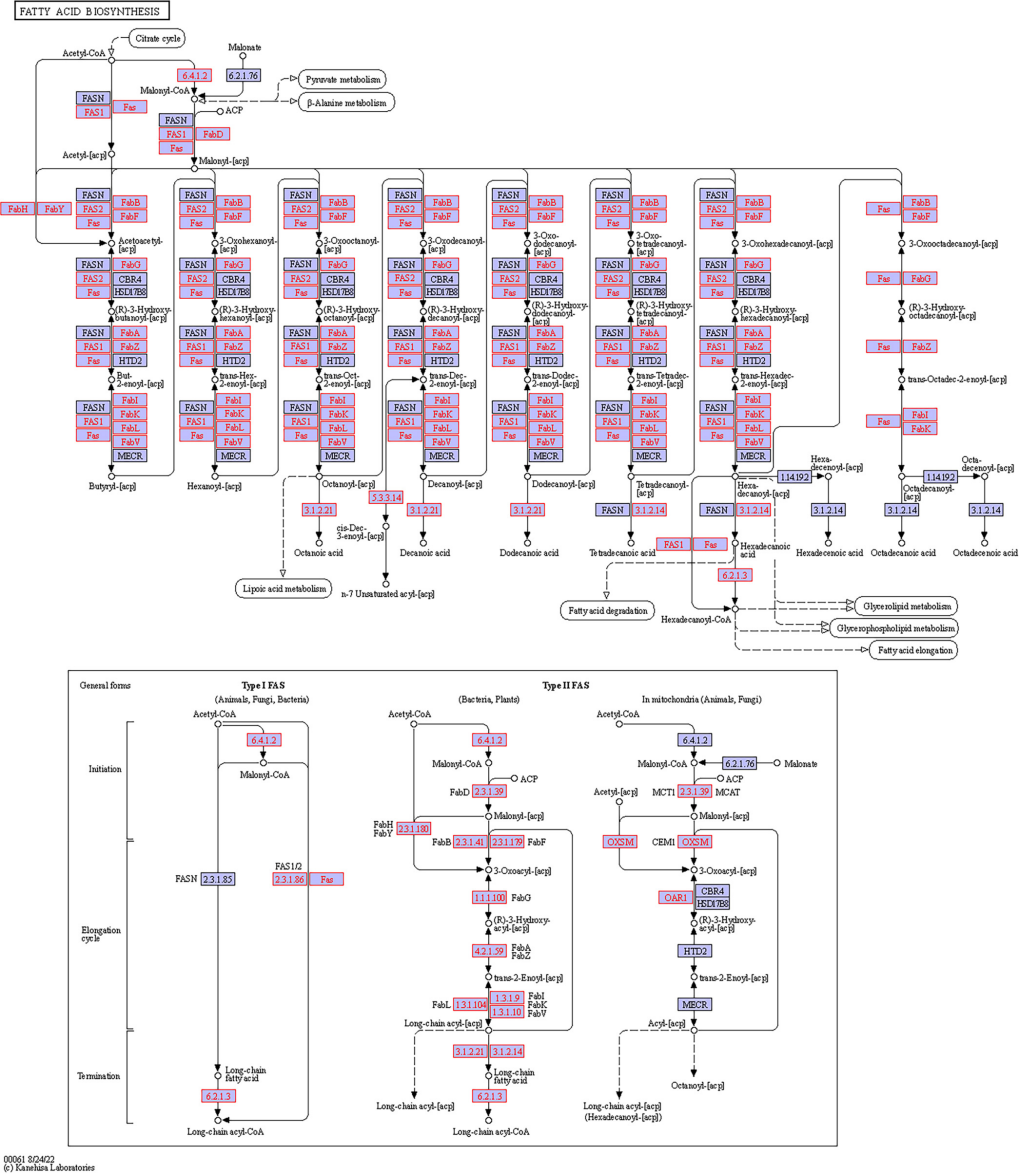
Hypothetical pathway for the biosynthesis of fatty acids by the gut microbial assembly of *T. gratilla elatensis*. The metabolic mapping was performed according to KEGG. Purple boxes with red font represent the genes that have been identified, while purple boxes with black font represent other known genes in the hypothetical pathway of each compound (marked by circles). Arrows present the direction of the metabolic function. Abbreviations: ACP, acyl carrier protein; FAS, fatty acid synthase bacteria type; FASN, fatty acid synthase animal type; MECR, mitochondrial enoyl-[acyl-carrier protein] reductase.

**FIG 11:**
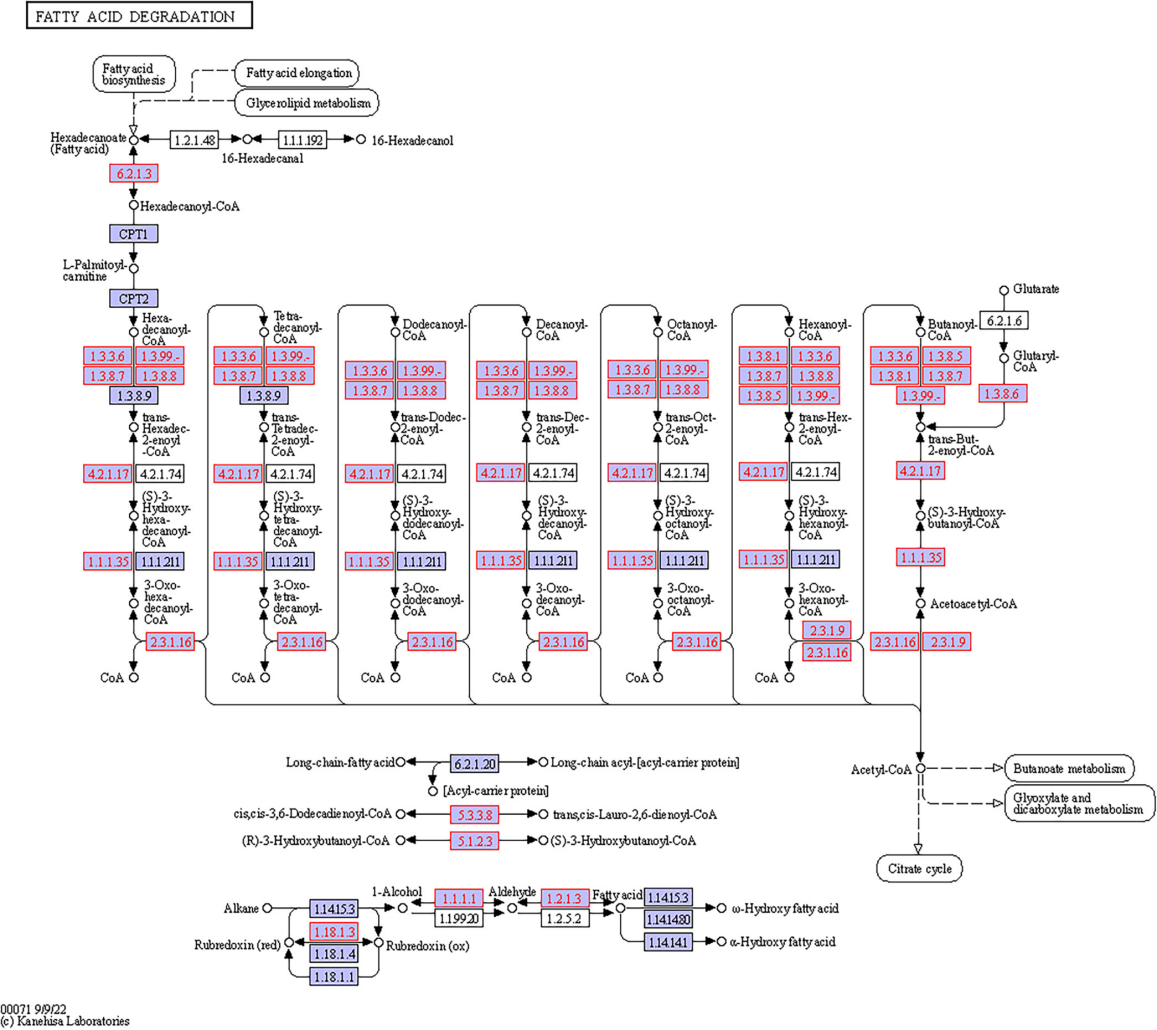
Hypothetical pathway for the degradation of fatty acids by the gut microbial assembly of *T. gratilla elatensis*. The metabolic mapping was performed according to KEGG. Purple boxes with red font represent the genes that have been identified, while purple boxes with black font represent other known genes in the hypothetical pathway of each compound (marked by circles). Arrows present the direction of the metabolic function.

A total of 233 KOs were collected from the data and demonstrated five bacterial metabolic pathways in the gut. Sixty-nine KOs were involved in starch and sucrose metabolism, 57 in sulfur metabolism, 41 in nitrogen metabolism, 41 in fatty acid metabolism, and 25 in fatty acid degradation (Fig. 6). All of these KOs were present both in the stomach and in the intestine, and many of them (222) also appeared in the esophagus. However, the dissimilarity between the different gut regions was significant (*T* = 9.58, *P* < 0.0001) due to differences in the number of copies of the various KOs (Table S2). Specifically, 85 KOs related to various metabolisms revealed a significant variation in their number of copies in the different regions (Tables S3 and S4). Nearly all (83 KOs) had more copies in the intestine than the esophagus, while 74 KOs had more copies in the intestine than in the stomach or the esophagus. The 69 KOs of the starch and sucrose metabolism formed modules for the metabolism of cellulose, cellobiose, levan, starch, and sucrose. All modules consisted of genes for both the biosynthesis and degradation of these polysaccharides (Fig. 7). For sucrose and starch metabolism, the esophagus contained fewer gene copies than did the stomach or intestine (*F* = 21.83, *P* < 0.0001). Sulfur metabolism modules included thiosulfate oxidation to sulfate (S_2_O_3_ to SO_4_, i.e., the Sox pathway) and reduction of sulfate to sulfide (SO_4_ to H_2_S) through assimilatory or dissimilatory metabolism (Fig. 8). The esophagus contained more gene copies for Sox metabolism (*F* = 590.4, *P* < 0.0001), but the stomach and intestine had more gene copies for assimilatory reduction of sulfate to sulfide (*F* = 16.6, *P* < 0.0001). Modules for nitrogen metabolism included N fixation (N to NH_3_), nitrification (NH_3_ to NO_3_), and denitrification (NO_3_ to N_2_), as well as assimilatory and dissimilatory reduction of nitrate to ammonia (NO_3_ to NH_3_) (Fig. 9). More gene copies for N fixation were found in the esophagus than in the other gut regions (*F* = 7.988, *P* < 0.05). The genes identified for fatty acid biosynthesis formed modules for initiation, elongation, and termination of the biosynthesis of long-chain saturated and unsaturated fatty acids (Fig. 10). The degradation of these fatty acids is enabled by the presence of KOs that are involved in all of the distinguished modules for such metabolism (Fig. 11). The number of gene copies for either the synthesis or the degradation of fatty acids was higher in the intestine than in the esophagus (*F* = 14.06/4.306, *P* < 0.0001/0.05, respectively).

## DISCUSSION

Spatial variations in the gut microbial community of *T. gratilla elatensis* can aid our understanding of microbes’ contribution to their host in food digestion. Such changes underlie a substantial dissimilarity between the distal ends of the digestive tract, i.e., the anterior esophagus and posterior intestine. The intermediate bacterial community in the stomach is somewhat more similar to that in the intestine, as both contribute to food digestion and fermentation. Regional specialization of some resident bacteria in the gut can be associated with their local environmental conditions and their contribution to food digestion and other metabolisms.

The alterations in the compositional and functional structures of the microbial communities in the esophagus, stomach, and intestine regions can be attributed to differences between their anatomical and physiological characteristics. The esophagus in sea urchins is characterized by a low retention time of the ingested feed, up to a few hours, and a high interchange with the surrounding seawater outside the body (8). Such high contact between the internal esophagus and the external environment resulted in a bacterial community that was poor and less diverse but consisted of 9 taxonomic groups that were unique to this region. These unique groups included the *Mangrovimonas*, *Lachnospiraceae*, *Hyphomicrobiaceae*, *Rhizobiales*, *Roseibium*, *Shimia*, *Legionella*, *Nesiotobacter*, and *Rhodobacterales*. We propose the esophagus as a more transitory niche in the digestive tract of *T. gratilla elatensis*. With a low content of nutrients and a high exchange rate of bacteria, the diversity in the esophageal bacterial community was relatively low and may have contained many bacterial outlanders that do not necessarily contribute to food digestion (20). The relatively high number of gene copies for Sox metabolism in the esophagus may be attributed to the high water exchange rate and aerobic conditions in this region, as also revealed in another sea urchin species (16). However, this region is still an essential transport station for the bacteria resident in the stomach and intestine, which are necessary to the host for their involvement in food digestion.

In contrast, the stomach and intestine are proposed as the settlement areas of biodiverse communities with many food-digesting and fermenting bacteria. These regions offer the resident bacteria more determinate niches with specific environmental conditions regarding the abiotic parameters and available nutrients. Previous studies on sea urchins (i.e., Echinocardium cordatum, Paracentrotus lividus, and Spatangus purpureus) revealed the stomach as an extreme environment with a relatively acidic pH, low oxygen, high carbon dioxide, and elevated concentrations of short-chain fatty acids (SCFAs) (11, 21, 22). Bacterial fermentation has been proposed to increase SCFAs and reduce pH levels, actions which are essential in breaking down food particles in this region (11, 20).

In the intestine, pH and oxygen increase to levels similar to those in the far interior esophagus. However, significant differences between these parts under local conditions may include a higher level of sulfate, sulfide, organics, and SCFAs in the intestine, where food digestion and fermentation continue (11). Our findings support rapid bacterial activity in various functions and metabolic processes in the intestine region compared to the esophagus.

The occurrence of bacteria of the genus “*Candidatus* Hepatoplasma” in all the examined regions agrees with the results of earlier studies that proposed “*Ca*. Hepatoplasma” as a core taxon in the gut of sea urchins *T. gratilla elatensis* and Lytechinus variegatus (17, 19, 23). Other studies considered members of “*Ca*. Hepatoplasma” as either symbionts or parasites in the gut of other marine organisms like arthropods and isopods (24, 25), supported by the characterization of their relatively small bacterial genome (26, 27). The higher abundance of “*Ca*. Hepatoplasma” in a nutrient-depleted region such as the esophagus may support its host-dependent lifestyle in *T. gratilla elatensis*. In any case, it strengthens the probability of finding this taxon in this sea urchin’s gut.

The conclusions in this current research support the primary contribution of the stomach and intestine bacteria in food digestion, fermentation, and metabolism of the residuals of such processes. Significant evidence of this is the increased level of bacterial genes related to energy production in the metabolism of carbohydrates, sugars, and fatty acids in these regions, with the highest levels found in the intestine.

Among the obtained taxa in this region, we propose the phyla *Bacteroidetes* and *Firmicutes* (also referred to as *Bacillota*) as the main bacterial phyla in food digestion and fermentation processes. The former two taxa succeeded and predominated in the posterior regions of the stomach and intestine, while all presenting features associate them with the processes mentioned above. *Bacteroidetes* and *Firmicutes* are known for their cellulolytic activity in the decomposition of plant biomass (28–31). Their high abundance in the posterior regions of the gut of *T. gratilla elatensis* (i.e., stomach and intestine) may be efficient under the current dietary regime for the decomposition of the cellulose-rich *Ulva.* Moreover, the high number of amylase-encoding genes in the stomach of *T. gratilla elatensis* agrees with previous studies that identified a high activity of β-glucuronidase in the stomach of five species of sea urchin (8). The dominance of *Bacteroidales* in the stomach also supports the metabolism of carbohydrates in this region (31). Moreover, several members of the *Bacteroidales* were identified as core microbes, i.e., present in the gut of this species regardless of the diet type (18).

Sea urchins are considered poor in digestive enzymes with endogenic amylases, proteases, and lipases (7, 8, 10), hence the importance of bacterial contribution to food digestion. A primary outcome of the polysaccharide decomposition activity is the formation of simple sugars used in bacterial fermentation. The latter may be crucial for gaining energy from the polysaccharide-rich *Ulva.* It was estimated that bacterial fermentation products in the gut might provide the sea urchin with 10% of the energy required for its metabolism (20). In the current research, the gut bacterial community of *T. gratilla elatensis* was rich in fermentation-related genes of fatty acid metabolism, including SCFA production. Among the identified gut bacteria, members of the *Lachnospiraceae* (*Bacillota*; *Clostridiales*) are efficient fermenters that produce SCFAs from plant polysaccharides in ruminants (32, 33) and humans (34). A single ASV of this taxon was identified here but only in the stomach. Of the phylum *Firmicutes*, *Ruminococcaceae* were a unique taxonomic group in the intestine of *T. gratilla elatensis*, with no members in other regions. This unique taxon is considered a highly efficient fermenter that produces SCFAs such as butyrate, formic acid, and d-lactate (32). Propionate is a common precursor fatty acid for SCFA production, while various *Bacteroidetes* have been shown to produce it in their lipid metabolism through the succinate pathway (35, 36).

Regarding carbohydrate metabolism, it should be noted that *Ulva* is rich in sulfated polysaccharides like ulvans. Their content may reach 13% of the total saccharide content (37). The decomposition of these polysaccharides will therefore result in the release of the residual sulfates (12). In the current research, we reveal the potential pathways for bacterial reduction of the excess sulfate in the posterior regions of the gut of *T. gratilla elatensis*, mainly in the intestine. We propose the contribution of *Desulfotalea*, *Desulfitispora*, and *Defluviitalea* to sulfate reduction via assimilatory and dissimilatory mechanisms. A previous study also identified sulfate-reducing bacteria (SRB) of *Desulfotalea* in the intestine of the sea urchin *Paracentrotus lividus* (20). Although sulfate reduction requires anaerobic conditions, such activity was also measured in the intestinal cecum of another sea urchin (11, 38). The excess fermentation products in the stomach and intestine of sea urchins can support SRB as an energy source for this metabolism (20). The sulfide production by SRB may have facilitated bacterial oxidation of sulfide in the intestine, for example, by some of the identified alphaproteobacteria. This agrees with earlier studies proposing that members of that taxon are responsible for sulfide oxidation in the gut of other sea urchins (20, 39).

From an ecological perspective, we conclude that environmental conditions and functional specialization are influential deterministic factors that shape the bacterial community along the digestive tract. A deterministic effect of environmental conditions on gut bacteria has been reported in various animals. In dairy cattle (Bos taurus), alterations in bacterial community were associated with the extremely low pH in the rumen (40). A similar effect was associated with low oxygen in the mammalian lumen (41). In the current study, the composition and content of functional genes in the bacterial communities of the stomach and intestine overlapped. This result is unlikely, considering the significant differences in oxygen levels in these regions (i.e., depleted in the stomach and rich in the intestine). Moreover, dissimilarity was measured only between the esophagus and intestine bacterial communities, although these regions present relatively similar levels of oxygen. We therefore assume that bacterial functionality in fermentation is responsible for the low oxygen conditions in the stomach, as was also proposed in other sea urchins like *Echinocardium cordatum* or *Paracentrotus lividus* (11, 21). The theory on the function of the sea urchin’s gut as a continuous-flow stirred reactor also supports the overlap between the stomach and intestine microbiomes due to the high retention time of food and its potential mixing in these regions. Altogether, we conclude a significant contribution of the stomach and intestine bacteria to food digestion and energy gain through the decomposition of dietary polysaccharides, removal of the sulfate groups that are released in this process, and production of SCFAs via fermentation.

## MATERIALS AND METHODS

### Sea urchin culture and sampling.

Nine T*. gratilla elatensis* individuals were collected from the National Center for Mariculture (NCM) aquaculture unit on the Gulf of Aqaba. After body weight and diameter measurements, sea urchins were randomly distributed into three 90-L plastic tanks (0.67 by 0.48 by 0.34m), each containing three individuals. The experiment took place over 8 weeks. The sea urchins were fed *ad libitum* with *Ulva fasciata* from the local seaweed culture system following a recommended daily feeding protocol (42). This protocol yielded a sufficient growth rate of 0.2% day^−1^, with no mortality or morbidity. Seawater from the Gulf of Aqaba was pumped from the station on the northern shore, conveying seawater from a depth of 13 m, 300 m offshore (32°29′N and 58°34′E). Seawater constantly flowed through the culture tanks to provide sea urchins with the ambient abiotic conditions of the Red Sea and high water quality. The ambient temperature, salinity, and pH in the culture tanks during the experimental period were 26.5 ± 0.5°C, 4 ± 0.1%, and 8.15 ± 0.05, respectively. At the end of the culture period, five sea urchins were randomly removed from the tanks and dissected to sample their guts, and fecal leftovers from the tank were collected using a vacuum pump. The digestive tract was carefully removed using sterile scissors, ingesta was removed, and the gut tissue was separated into the three segments of esophagus (closest to the mouth-like organ known as Aristotle’s lantern), stomach (middle section), and intestine (last region before the rectum) (see Fig. S1 in the supplemental material). In our examined individuals, the mean length of the digestive tract was 20 cm, with mean lengths of 3, 11, and 6 cm for the esophagus, stomach, and intestine, respectively (Fig. S4). In order to prevent overlaps between the different gut parts during the dissection, we sampled the central region in each of these regions. Samples were placed in sterile Eppendorf tubes, dipped in liquid nitrogen for several seconds, and stored at −80°C until DNA extraction.

### DNA extraction, amplification, and amplicon sequencing.

The tissue samples were homogenized in a FastPrep-24 5G homogenizer (MP Biomedicals, CA, USA). Bacterial genomic DNA was purified from 0.2 g of the homogenate using the PureLink microbiome DNA purification kit and the recommended protocol for high-quality microbial DNA extraction from stool samples (Thermo Fisher Scientific, MA, USA). DNA quantitation and quality control were performed using a Qubit 3.0 fluorometer (Thermo Fisher Scientific, MA, USA). The 16S rRNA gene fragments were amplified from the DNA using the primers 515F and 926R for the V4-V5 variable regions, following previous recommendations (43). The PCR amplification was carried out on the template DNA (50 ng μL^−1^), using the abovementioned primers (0.5 μM each) and DreamTaq Green master mix consisting of MgCl_2_ (2 mM). The amplification program included denaturation at 95°C for 5 min, and 28 cycles each of 45 s at 94°C, 60 s at 50°C, and 90 s at 72°C, and the final extension at 72°C for 10 min. PCR products were analyzed by gel electrophoresis (Tris-borate-EDTA/agarose, 2.0% [wt/vol]) and stored at −20°C until sequencing. After measurement of the DNA concentration using a Qubit 3.0 fluorometer and diluting the concentration to 4 nM (in each sample), amplicon sequencing of the 16S rRNA genes was performed using the Illumina MiSeq platform (Illumina Inc., San Diego, CA, USA) (44) at the University of Illinois Research Resources Center (RRC).

### Data processing and statistical analyses.

The sequencing data were analyzed using the Quantitative Insights into Microbial Ecology pipeline (QIIME; version 2) (45). This included the removal of primers and linkers, merging pair-end reads, and filtering out sequences with a quality score under 33 or shorter than 300 bp. Sequences were clustered into amplicon sequence variants (ASVs) at a 97% similarity cutoff using UCLUST (46) and the Greengenes database while removing chimera sequences using the ChimeraSlayer tool (47). Taxonomy assignments were collected using the DADA2 classifier. The sequence’s rarefaction at a minimum depth of 1,167 reads was sufficient for data analyses in the MicrobiomeAnalyst software (48), i.e., calculating relative abundance and measuring the communities’ ecological indices (e.g., alpha and beta diversity). The bacterial community of each gut region was analyzed using five biological replicates from different sea urchin individuals. Differences in the composition of microbial assemblies were measured by Bray-Curtis dissimilarity and by using the weighted UniFrac (49). Permutational multivariate analysis of variance (PERMANOVA) and the Kruskal-Wallis test verified differences in bacterial communities in the different gut regions. The analyses of functional genes were performed after transferring the ASV data to PICRUSt2 software (50) for predicting the metagenomic content of functional genes in the different gut regions. Each identified functional gene received an Enzyme Commission (EC) number, and the functional genes were sorted into known functional categories in the KEGG (51) and COG (52) databases. The data on gene orthologues in the different gut regions were normalized using total sum scaling in which the count of each orthologous gene is divided by the total library size to yield a relative proportion of counts. This step eliminated technical biases associated with the various sequencing depths in different library sizes. Two-way analysis of variance measured differences in the abundance of the orthologous gene between the different gut regions. This analysis was performed to identify significant differences in the number of gene copies considering their annotation in the different general functional categories in the KEGG and COG databases and further on to identify significant differences in the number of gene copies for selected functional genes that were associated with food digestion and metabolism. The Kruskal-Wallis test was used to measure the statistical differences in the Shannon index of functional diversity between the communities in the different gut regions. Group similarity analyses (ANOSIM) were used to measure the differences in the functional beta diversity.

### Data availability.

The data sets generated in this study are accessible through ENA with the accession number PRJEB42313.
