# Supplementary material for: Spatial Succession Underlies Microbial Contribution to Food Digestion in the Gut of an Algivorous Sea Urchin
Source: Microbiol Spectr. 2023 Apr 25;11(3):e00514-23. doi: 10.1128/spectrum.00514-23 (PMC10269587; doi:10.1128/spectrum.00514-23)
Supplement: Supplemental file 1 — Fig. S1 to S4 and Tables S1 to S4. Download spectrum.00514-23-s0001.docx, DOCX file, 1.7 MB [file spectrum.00514-23-s0001.docx]

**Supplementary material**


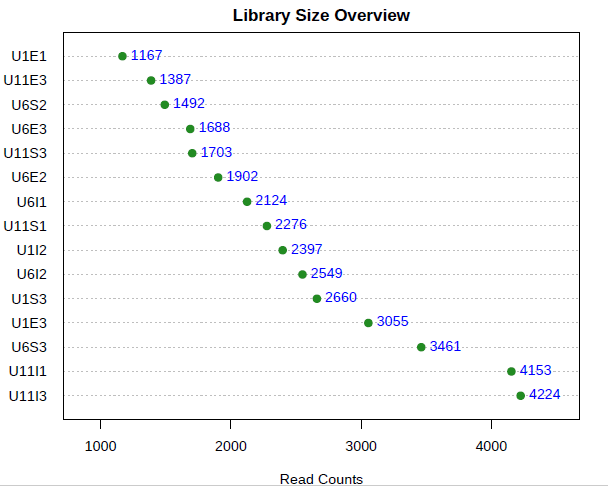
**Figures**

FIG. S1. The number of high-quality reads used for analyses of the gut microbial assembly in *T. gratilla elatensis*. The sample was categorized by diet and gut region followed by Sample ID.


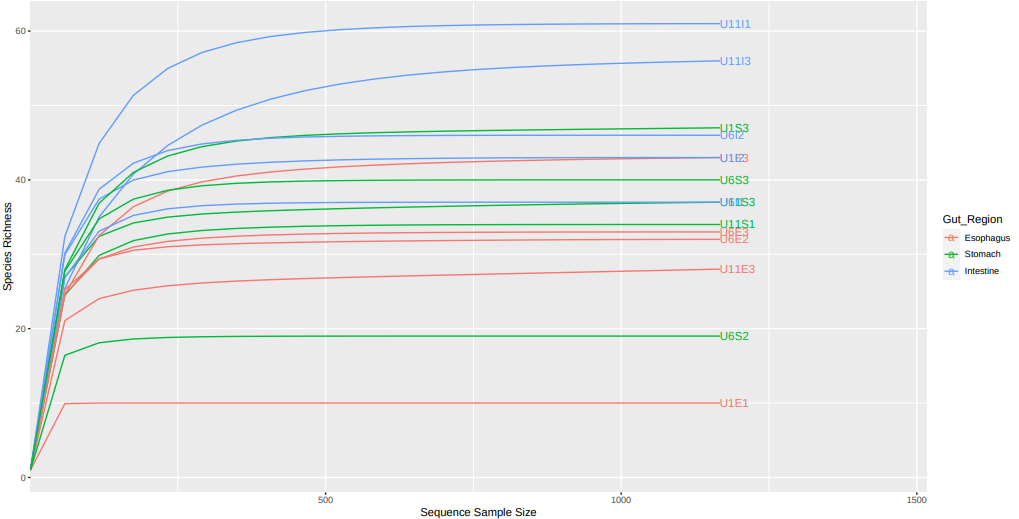


FIG S2 Species richness of the gut microbial assembly of *T. gratilla elatensis*. Rarefaction curves normalized to minimum library size present the number of ASVs from each of the samples and are colored by gut regions.


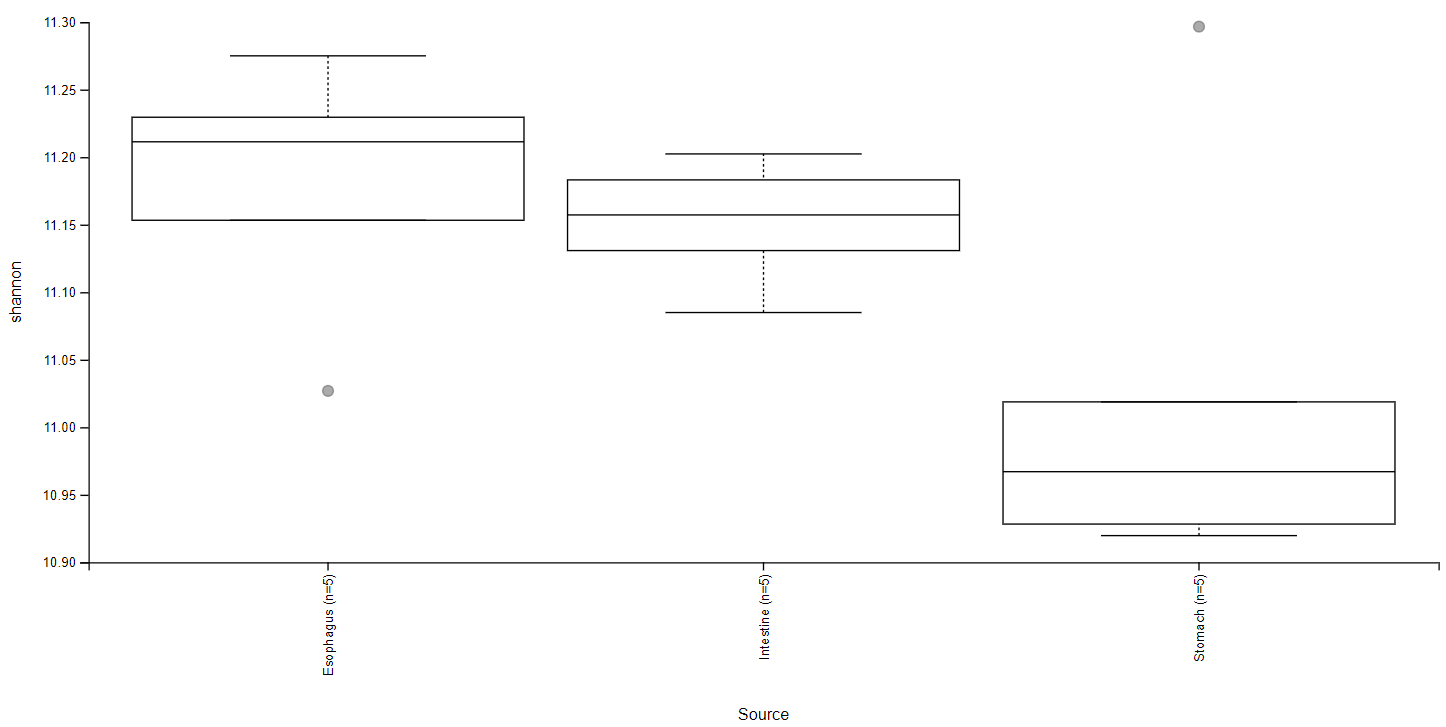
FIG S3 KEGG metabolism diversity of the gut microbial assembly of *T. gratilla elatensis.* Shannon index of diversity was presented with the attribution to gut region.

**
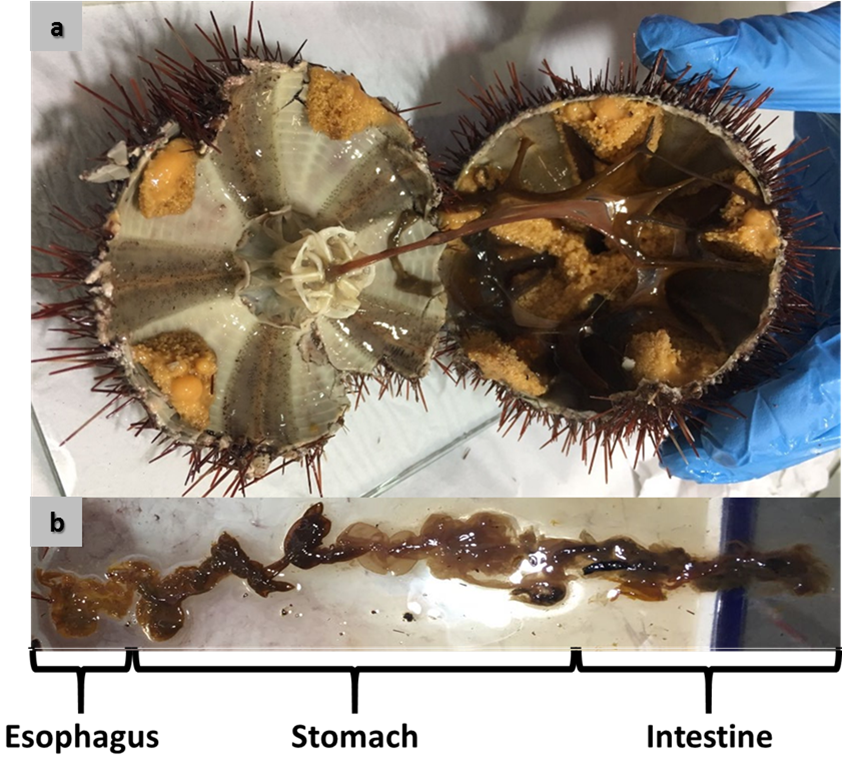
**

FIG S4 The inner body and the digestive tract of Tripneustes gratilla elatensis (a) A lateral view of the body's upper (left) and lower (right)
sides reveals the coelom's different organelles; (b) A spread gut after
dissection and separation from the body reveals the different gut part of
the esophagus, stomach, and intestine. Not to scale.

TABLE S1 Statistical analyses of the two-way ANOVA test for the hypothetical KEGG orthologue groups in the microbial assemblies of the different gut regions of *T. gratilla elatensis*.

TABLE S2 A pair-wised statistical analysis of all the identified KEGG orthologues (KOs) in the gut bacterial community of *T. gratilla elatensis.* The different gene orthologues were examined for statistical differentiation in the number of copies between each pair of gut regions. Values of the level of difference, i.e., ∆ between the mean number of copies in each of the pair-examined regions and the p-value, are presented. Positive values of the level of difference identify a higher number of gene copies in the 'reference' region (ref) while negative values identify a higher number of gene copies in the 'case' region (case). Gene orthologues that presented a significant difference between pair-examined regions are bolded. The list of KOs is sorted according to the different bacterial metabolism categories.

TABLE S3 Bacterial gene orthologues that revealed a significant difference between one or more of the pair-examined regions. The number of copies in each region is identified under the columns of that gut region’s name. The statistical analyses of the level of difference, i.e., ∆ between the mean number of copies in each of the pair-examined regions, is provided at the right. Positive values of the level of difference identify a higher number of gene copies in the 'reference' region (ref) while negative values identify a higher number of gene copies in the 'case' region (case). Gene orthologues that presented a significant difference between pair-examined regions are bolded. The list of KOs is sorted according to the different bacterial metabolism categories.

TABLE S4 List of KEGG annotations representing bacterial genes that contribute to different metabolisms in the gut of *T. gratilla elatensis.* Each row in the table provides data on the KEGG orthologue number (ko number); gene name; functionality of the encoded enzyme; and the enzyme commission number (EC). KEGG orthologues are listed according to the different bacterial metabolic categories.
